# Hsc70 rescues the synaptic vesicle trafficking defects caused by α-synuclein dimers

**DOI:** 10.17912/micropub.biology.000737

**Published:** 2023-03-01

**Authors:** Emily B. Brady, Molly McQuillan, Audrey T. Medeiros, Luigi Bubacco, Rui Sousa, Eileen M. Lafer, Jennifer R. Morgan

**Affiliations:** 1 The Eugene Bell Center for Regenerative Biology and Tissue Engineering, Marine Biological Laboratory, Woods Hole, Massachusetts, United States; 2 Department of Biology, Duke University, Durham, North Carolina, United States; 3 Neuroscience Graduate Program, Brown University, Providence, Rhode Island, United States; 4 Department of Biology, University of Padua, Padua, Veneto, Italy; 5 Department of Biochemistry and Structural Biology, The University of Texas Health Science Center at San Antonio, San Antonio, Texas, United States

## Abstract

Aberrant buildup of α-synuclein is associated with Parkinson’s disease (PD) and other neurodegenerative disorders. At synapses, α-synuclein accumulation leads to severe synaptic vesicle trafficking defects. We previously demonstrated that different molecular species of α-synuclein produce distinct effects on synaptic vesicle recycling, and that the synaptic phenotypes caused by monomeric α-synuclein were ameliorated by Hsc70. Here, we tested whether Hsc70 could also correct synaptic deficits induced by α-synuclein dimers. Indeed, co-injection of Hsc70 with α-synuclein dimers completely reversed the synaptic deficits, resulting in synapses with normal appearance. This work lends additional support for pursuing chaperone-based strategies to treat PD and other synucleinopathies.

**Figure 1.**
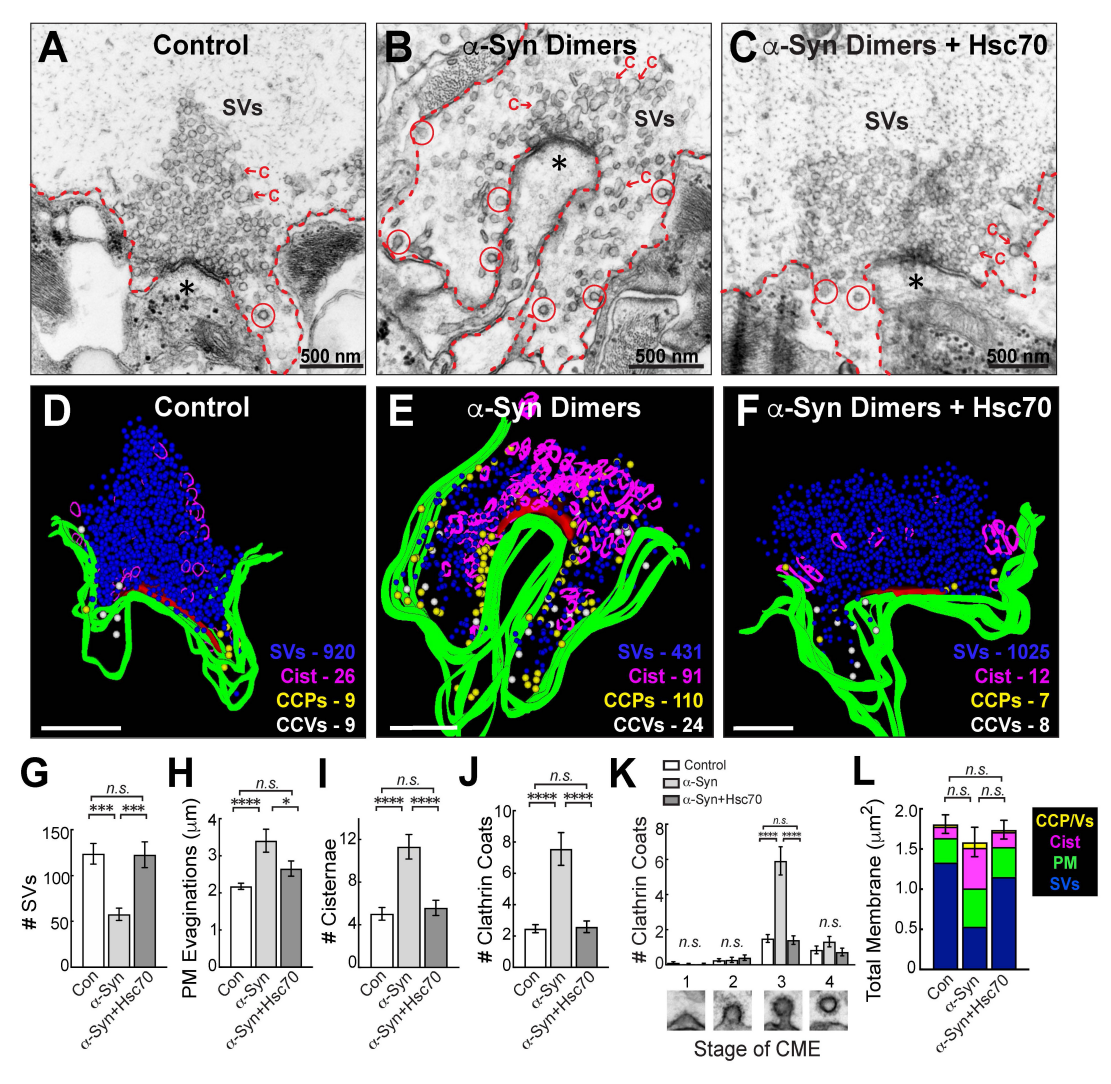
**Hsc70 rescues the endocytic phenotype caused by acute introduction of dimeric α-synuclein. (A)** Electron micrograph showing a control, stimulated synapse with a large synaptic vesicle (SV) cluster, shallow plasma membrane (PM) evaginations (dotted lines), and only a few cisternae (C). **(B)** In comparison, synapses treated with dimeric human α-synuclein had small SV clusters, large PM evaginations, and increased numbers of cisternae and clathrin-coated pits (CCPs; red circles). This phenotype is consistent with an inhibition of the fission step during clathrin-mediated SV recycling. **(C)** Co-injection of recombinant Hsc70 reverses these effects. In panels A-C, the asterisks indicate postsynaptic dendrites. **(D-F)** 3D reconstructions of the synapses in A-C, which were generated from a series of 5 ultrathin sections using Reconstruct software (Fiala, 2005). SVs were rendered as blue spheres; cisternae/endosomes as magenta ribbons; plasma membrane evaginations as green ribbons; and CCPs (clathrin-coated pits) or CCVs (free clathrin-coated vesicles) as yellow or white spheres, respectively. **(G-L)** Morphometric analysis revealed that co-injection of Hsc70 with α-synuclein dimers reverses the loss of SVs and reduces the expansion of PM evaginations, as well as cisternae and CCPs induced by excess dimeric α-synuclein. In all cases, the presence of Hsc70 restored the synaptic organelles and membranes to control values. Bars represent mean +/- SEM from n=22-24 synapses, 2 axons/animals. * indicates p<0.05; *** indicates p<0.001; **** indicates p<0.0001, and “ *n.s.* ” indicates “not significant” by ANOVA.

## Description

α-Synuclein is a synaptic vesicle-associated protein with multi-faceted roles in exocytosis and endocytosis at synapses (Sulzer and Edwards, 2019; Sharma and Burre, 2022). Although its normal functions are still being investigated, it is widely accepted that aberrant accumulation and aggregation of α-synuclein are associated with a variety of neurodegenerative diseases such as Parkinson’s disease (PD), Dementia with Lewy Bodies (DLB), and several variants of Alzheimer’s disease (AD) (Sulzer and Edwards, 2019; Sharma and Burre, 2022). Atypical accumulation of α-synuclein at synapses causes severe synaptic vesicle (SV) trafficking defects and is now thought to represent a significant early step in disease pathogenesis in both animal models and human patients (Nemani et al., 2010; Schulz-Schaeffer, 2010; Scott et al., 2010; Busch et al., 2014). Thus, two major goals are to determine how excess α-synuclein causes such synaptic deficits and to identify effective strategies for amelioration, which are currently lacking.

Using the lamprey giant reticulospinal synapse as a model, we previously demonstrated that acute introduction of excess human α-synuclein inhibited SV recycling, including impacts on clathrin-mediated endocytosis (CME) and bulk endocytosis (Busch et al., 2014; Medeiros et al., 2017; Banks et al., 2020; Soll et al., 2020). This perturbation led to a reduction in the size of SV clusters, compensated by an expansion of plasma membrane evaginations and increased numbers of endocytic intermediates such as clathrin-coated vesicles (CCVs) and endosome-like vesicles. Similar impacts on SV endocytosis were observed at the mammalian calyx of Held synapse, where additionally it was shown that excess α-synuclein had minimal impact on exocytosis (Xu et al., 2016; Eguchi et al., 2017). While excess monomeric α-synuclein impaired the uncoating of CCVs, dimeric a-synuclein impaired the fission of clathrin-coated pits (CCPs) from the plasma membrane (Medeiros et al., 2017; Banks et al., 2020). Moreover, physiological α-synuclein multimers (60-100 kDa) consistent with tetramers and related species had no obvious effects on CME but instead impaired intracellular vesicle trafficking, leading to an abundance of endosome-like vesicles (Roman-Vendrell et al., 2021). Thus, different oligomeric species of α-synuclein impact distinct stages of activity-dependent SV trafficking, raising new questions about how these different phenotypes can be corrected.

Molecular chaperone proteins such as Hsc70 are emerging as potential ameliorators of α-synuclein-induced neuronal toxicity, including at synapses. Hsc70 directly interacts with α-synuclein *in vitro* and in living cells (Pemberton and Melki, 2012; Redeker et al., 2012; Banks et al., 2020; Burmann et al., 2020). Hsc70, Hsp90, and several other heat shock proteins are abundant in Lewy bodies in PD, DLB, and Lewy Body Variant of AD brains (Auluck et al., 2002; Uryu et al., 2006). Overexpression of several chaperones reversed α-synuclein aggregation and pathology in both *Drosophila* and mouse models (Auluck et al., 2002; Taguchi et al., 2019). Recent mechanistic studies indicate that Hsc70 disaggregates α-synuclein aggregates in cells (Schneider et al., 2021). At lamprey synapses, the SV recycling defects caused by excess monomeric α-synuclein were ameliorated by co-injection with Hsc70, including a reversal of the CCV uncoating defect (Banks et al., 2020). Thus, there are now multiple contexts and cell types in which molecular chaperones have been shown to reduce α-synuclein toxicity.

Building on our prior work at lamprey synapses, we set out to determine whether Hsc70 could also rescue the SV trafficking defects caused by acute introduction of α-synuclein dimers to synapses. Stable recombinant human α-synuclein dimers were generated by covalent linkage of two α-synuclein molecules at their C-termini, as described in prior studies (Pivato et al., 2012; Medeiros et al., 2017). Dimeric α-synuclein (50 μM in pipet) was microinjected into lamprey giant reticulospinal axons, thereby directly delivering the protein to the presynapses. After dilution and diffusion in the axon, we estimate the final axonal concentration of dimeric α-synuclein to be ~3-5 μM, which is ~1-2X the best estimates of α-synuclein concentration at synapses and commensurate with the mild overexpression levels observed in PD (Singleton et al., 2003; Westphal and Chandra, 2013; Wilhelm et al., 2014). Injected axons were then stimulated (20 Hz, 5 min) before fixing and processing the spinal cords for electron microscopy. Images of control synapses were taken from the injected axon, but from a location beyond where the protein had diffused. As expected, control synapses had a normal appearance with large vesicle clusters, small plasma membrane evaginations, and very few “cisternae” (putative endosomes) or CCP/Vs **(Figure 1A, D)** . In contrast, synapses treated with α-synuclein dimers had smaller vesicle clusters, large plasma membrane evaginations, numerous cisternae, and increased numbers of CCPs attached to the plasma membrane, consistent with the impairment of clathrin-mediated synaptic vesicle recycling and fission defects that we previously reported **(Figure 1B, E)** (Medeiros et al., 2017). Upon co-injection of substoichiometric amounts of recombinant bovine Hsc70 (~1-3 μM), α-synuclein dimers no longer caused major impacts on SV endocytosis, and the synaptic morphology appeared largely normal **(Figure 1C, F)** . Thus, Hsc70 appeared to rescue the α-synuclein-induced synaptic defects, as shown clearly by the 3D reconstructions of treated synapses **(Figure 1D-F)** .

To quantify these effects, we performed a morphometric analysis on the synaptic membranes. Indeed, co-injection of Hsc70 completely rescued the loss of SVs induced by α-synuclein dimers ( **Figure 1G;** Control: 124 ± 11 SVs/section; α-Synuclein: 58 ± 7 SVs; α-Synuclein+Hsc70: 123 ± 14 SVs; n=22-34 synapses, 2 axons/condition; p<0.0001 ANOVA). Similarly, Hsc70 reversed the expansion of the plasma membrane caused by α-synuclein dimers ( **Figure 1H;** Control: 2.2 ± 0.1 μm; α-Synuclein: 3.4 ± 0.3 μm; α-Synuclein+Hsc70: 2.7 ± 0.2 μm; n=22-34 synapses, 2 axons/condition; p=0.0001 ANOVA). Likewise, the number of cisternae were restored to control levels with Hsc70 ( **Figure 1I;** Control: 5.0 ± 0.6 cisternae/section; α-Synuclein: 11.3 ± 1.2 cisternae; α-Synuclein+Hsc70: 5.6 ± 0.7 cisternae; n=22-34 synapses, 2 axons/condition; p<0.0001 ANOVA). The total number of clathrin coated structures (CCPs + CCVs) were also restored to normal levels ( **Figure 1J;** Control: 2.5 ± 0.3 clathrin coats/section; α-Synuclein: 7.6 ± 1.0 clathrin coats; α-Synuclein+Hsc70: 2.6 ± 0.4 clathrin coats/synapse, n=22-34 synapses, 2 axons/condition; p<0.0001 ANOVA), as was the distribution of clathrin coats at each stage of clathrin-mediated endocytosis **(Figure 1K)** . Importantly, the CME fission defects observed with dimeric α-synuclein were also corrected upon co-injection of Hsc70 **(Figure 1K, stage 3)** . Further analysis revealed that co-injection of Hsc70 completely reversed the altered membrane distribution induced by dimeric α-synuclein alone (i.e. the loss of SVs and increased PM, cisternae, CCP/Vs, indicative of endocytic deficits), with no change in total membrane **(Figure 1L;** Control: 1.8 ± 0.1 μm ^2^ ; α-Synuclein: 1.6 ± 0.2 μm ^2^ ; α-Synuclein+Hsc70: 1.8 ± 0.1 μm ^2^ ; n=22-34 synapses, 2 axons/condition; p=0.73 ANOVA). Thus, co-injecting Hsc70 with α-synuclein dimers resulted in a normal distribution of synaptic membranes.

The data presented here corroborate and extend prior findings that exogenous Hsc70 can ameliorate the SV trafficking defects associated with excess α-synuclein. Our prior study showed that co-injecting substoichiometric amounts of Hsc70 along with excess monomeric α-synuclein reversed the α-synuclein-induced impairment of SV trafficking, including impacts on CCV uncoating (Banks et al., 2020). In this study, we further demonstrate that Hsc70 can also rescue the SV recycling defects caused by α-synuclein dimers, including impacts on CCP fission **(Figure 1)** . Given that different oligomeric species of α-synuclein cause distinct impacts at synapses, this important finding raises the interesting possibility that Hsc70 may be a broad ameliorator of α-synuclein-induced synaptic deficits. Additional studies will be needed in order to determine the full extent to which Hsc70 can rescue the synaptic phenotypes caused by other molecular variants of α-synuclein and whether other modes of SV recycling besides the clathrin pathway are also affected. Nonetheless, these data provide additional support for the therapeutic potential of molecular chaperones in treating Parkinson’s disease and other synucleinopathies (Pemberton and Melki, 2012; Gorenberg and Chandra, 2017).

## Methods

***Spinal cord dissections and microinjections.*** Detailed methods for acute perturbations at lamprey synapses can be found in (Medeiros et al., 2017; Banks et al., 2020). All animal procedures were approved by the MBL Institutional Animal Care and Use Committee in accordance with NIH standards. Briefly, late larval stage lampreys ( *Petromyzon marinus* ) were anesthetized in 0.1 g/L MS-222 in tank water. Segments (2-3 cm) of lamprey spinal cords were dissected and pinned ventral side up in a Sylgard-lined petri dish in fresh, oxygenated lamprey ringer (in mM): 100 NaCl; 2.1 KCl; 1.8 MgCl _2_ ; 4 glucose; 2 HEPES, pH 7.4; 0.5 glutamine; 2.6 CaCl _2_ . Recombinant human α-synuclein dimers and bovine Hsc70 were generated as previously described (see Reagents) (Pivato et al., 2012; Sousa et al., 2016; Medeiros et al., 2017). Proteins were then diluted to their final pipet concentrations in lamprey internal solution (180 mM KCl, 10 mM HEPES, pH 7.4) before loading into glass microelectrodes. α-Synuclein dimers (50 μM pipet concentration), either alone or co-injected with Hsc70 (27 μM pipet concentration), were microinjected into lamprey giant reticulospinal axons using short puffs of nitrogen delivered via a picospritzer device (5-20 ms, 40 psi, 0.2-0.3 Hz). Injections lasted for 15-20 minutes, during which time the proteins diluted and diffused laterally within the axon. The estimated final axonal concentrations of the proteins were 3-5 μM dimeric human α-synuclein and 1-3 μM bovine Hsc70, as in our prior study (Banks et al., 2020). Axons were then stimulated at 20 Hz for 5 minutes using current injections (30-60 nA) before fixing immediately in 3% glutaraldehyde and 2% paraformaldehyde in 0.1 M Na cacodylate, pH 7.4 for at least 3 hours at room temperature and then overnight or longer at 4 ^o^ C.

***Electron microscopy.*** Fixed spinal cords were processed and embedded for standard electron microscopy, sectioned at 70 nm, and post-stained with uranyl acetate and lead citrate, as previously described (Busch et al., 2014; Medeiros et al., 2017; Banks et al., 2020; Soll et al., 2020). Images were obtained at 37,000X magnification using a JEOL JEM 200 CX electron microscope. Images of synapses treated with dimeric α-synuclein, or dimeric α-synuclein with Hsc70, were obtained at distances 20-140 μm away from the injection site. Control synapses were obtained at distances >400 μm away from the injection site, beyond which the proteins had diffused, thereby providing an internal control for all experiments. 3D reconstructions were generated from n=5 serial sections using Reconstruct software (Fiala, 2005). Morphometric analyses were performed on n=34 control synapses, n=25 α-synuclein dimer-treated synapses, and n=22 α-synuclein/Hsc70-treated synapses from n=2 axons/animals for each experimental condition. Measurements included the numbers of synaptic vesicles, cisternae, and clathrin-coated structures, as previously described (Busch et al., 2014; Medeiros et al., 2017; Banks et al., 2020). Plasma membrane evaginations were calculated by measuring the curved line along the plasmalemma out to a 1 μm distance from either edge of the synaptic active zone, and averaging the two measurements together for each synapse. For the total membrane analysis, SV and CCV surface areas were calculated by multiplying the surface area of a sphere (4πr ^2^ where r = d/2, and d=average diameter of the SVs) by the numbers of SVs or CCVs per synapse. Cisternae and PM areas were calculated by multiplying the summed perimeters of the cisternae or the length of PM evaginations, respectively, by the section thickness (70 nm).

***Statistical analyses.*** Graphing and statistical analyses were performed in GraphPad Prism 9 software. All image analyses were performed by researchers who were blinded to the experimental conditions. All data are reported as the mean ± SEM per section per synapse. Ordinary one-way ANOVA was performed for all datasets, followed by multiple comparisons. Threshold for significance was established using a p value <0.05. Results of the statistical analyses, including the p-values, are reported in the Results section and Figure 1.

## Reagents

**REAGENTS**

**Table:** 

**PROTEIN**	**SPECIES**	**EXPRESSION VECTOR**	**HOST**	**REFERENCES**
α-Synuclein	Human	pET28b	*E. coli* BL21 (DE3)	*Pivato et al., 2012* *Medeiros et al., 2017*
Hsc70	*Bos taurus*	pET28a+	*E. coli* BL21-CodonPlus (DE3) - RIPL	*Banks et al., 2020* *Sousa et al., 2016*
